# Genome-Wide Linkage Mapping Reveals QTLs for Seed Vigor-Related Traits Under Artificial Aging in Common Wheat (*Triticum aestivum*)

**DOI:** 10.3389/fpls.2018.01101

**Published:** 2018-07-27

**Authors:** Jinghong Zuo, Jindong Liu, Fengmei Gao, Guihong Yin, Zhi Wang, Fengying Chen, Xiaoying Li, Jimei Xu, Tiantian Chen, Lei Li, Yu Li, Xianchun Xia, Hong Cao, Yongxiu Liu

**Affiliations:** ^1^Key Laboratory of Plant Molecular Physiology, Institute of Botany, The Chinese Academy of Sciences, Beijing, China; ^2^College of Life Science, University of Chinese Academy of Sciences, Beijing, China; ^3^National Wheat Improvement Center, Institute of Crop Sciences, Chinese Academy of Agricultural Sciences, Beijing, China; ^4^Crop Research Institute, Heilongjiang Academy of Agricultural Sciences, Harbin, China; ^5^Zhoukou Academy of Agricultural Sciences, Zhoukou, China

**Keywords:** controlled deterioration, linkage analysis, longevity, 90K SNP array, seed storability, *Triticum aestivum*

## Abstract

Long-term storage of seeds leads to lose seed vigor with slow and non-uniform germination. Time, rate, homogeneity, and synchrony are important aspects during the dynamic germination process to assess seed viability after storage. The aim of this study is to identify quantitative trait loci (QTLs) using a high-density genetic linkage map of common wheat (*Triticum aestivum*) for seed vigor-related traits under artificial aging. Two hundred and forty-six recombinant inbred lines derived from the cross between Zhou 8425B and Chinese Spring were evaluated for seed storability. Ninety-six QTLs were detected on all wheat chromosomes except 2B, 4D, 6D, and 7D, explaining 2.9–19.4% of the phenotypic variance. These QTLs were clustered into 17 QTL-rich regions on chromosomes 1AL, 2DS, 3AS (3), 3BS, 3BL (2), 3DL, 4AS, 4AL (3), 5AS, 5DS, 6BL, and 7AL, exhibiting pleiotropic effects. Moreover, 10 stable QTLs were identified on chromosomes 2D, 3D, 4A, and 6B (*QaMGT.cas-2DS.2*, *QaMGR.cas-2DS.2*, *QaFCGR.cas-2DS.2*, *QaGI.cas-3DL*, *QaGR.cas-3DL*, *QaFCGR.cas-3DL*, *QaMGT.cas-4AS*, *QaMGR.cas-4AS*, *QaZ.cas-4AS*, and *QaGR.cas-6BL.2*). Our results indicate that one of the stable QTL-rich regions on chromosome 2D flanked by *IWB21991* and *IWB11197* in the position from 46 to 51 cM, presenting as a pleiotropic locus strongly impacting seed vigor-related traits under artificial aging. These new QTLs and tightly linked SNP markers may provide new valuable information and could serve as targets for fine mapping or markers assisted breeding.

## Introduction

Seeds during long-term storage will gradually deteriorate, lose vigor and stress resistance, and die ultimately (Nguyen et al., 2012; Waterworth et al., 2016). Poor germination resulted from seed deterioration may affect agricultural production such as non-uniform growth and reduced yield (Cantliffe et al., 1981; Schwember and Bradford, 2010). Seed aging process is inevitable during storage and it has been estimated that approximately 25% of the annual value will be lost owing to poor seed quality (McDonald, 1999; Rajjou et al., 2008; Schwember and Bradford, 2010), hence the importance of the assessment of seed deterioration to effective preservation in agriculture (Ahmed et al., 2016).

Progressive loss of seed vigor is often occurred during the seed storage (Rajjou et al., 2008; Bewley et al., 2013; Zhang et al., 2016). Studies of seeds storability under normal conditions take a long time to complete, so artificial aging or CDT have been applied to mimic natural aging (Bentsink et al., 2000; Rajjou et al., 2008). In CDT treatment, temperature and humidity are the crucial factors to modulate the degree of artificial aging (Delouche and Baskin, 1973; Bentsink et al., 2000; Zhang et al., 2016). Under artificial aging, germination of seeds with high vigor maintained relatively rapid and homogeneous, while low vigor seeds exhibited a marked decline (Landjeva et al., 2010; Han et al., 2014). Assessment of seed viability is another approach along with germination test (Smith et al., 2003; Ahmed et al., 2016). Time, rate, homogeneity, and synchrony are important aspects that needed to be measured during the dynamic germination process (Ranal and de Santana, 2006; Bewley et al., 2013).

Reactive oxygen species have been reported to be a major damage during seed aging (Hendry, 1993; Bailly, 2004; Borisjuk and Rolletschek, 2009). Lipid peroxidation resulting in the loss of cellular membrane integrity also causes damage to seed vigor (Priestley, 1986; Corbineau et al., 2002). In addition, energy metabolism, damage to RNA repair and protein synthesis, and DNA degradation are considered as factors affecting the loss of seed vigor during seed aging (Smith and Berjak, 1995; McDonald, 1999; Ahmed et al., 2016). Further, stress-related proteins also play a positive role in seed longevity (Prieto-Dapena et al., 2006; Kotak et al., 2007; Han et al., 2014).

Analysis of mutant or transgenic plant has revealed that many genes from various signaling pathways are involved in seed longevity. Mutations within the genes of *DOG1* (*DELAY OF GERMINATION1*), and *SNL1/2* (*SWI-INDEPENDENT3* (*SIN3*)*-LIKE*) in *Arabidopsis*, which led to reduced seed dormancy, are associated with a shortened seed longevity phenotype (Bentsink et al., 2006; Wang et al., 2013). This indicates that seed dormancy mechanisms might somehow cause delaying of seed deterioration. *Arabidopsis vte1* (*vitamin E deficient1*) and *vte2* mutants with tocopherol deficiency showed reduced seed longevity, which suggests that vitamin E is important for seed longevity by preventing lipid peroxidation (Sattler et al., 2004). Accumulation of constitutive reactive oxygen species in the seed of *ferric-chelate reductase1*, a mutant with the function of mitochondrial NADH dehydrogenase, was found sensitive to aging (Clerkx and Koornneef, 2003; Bueso et al., 2014). Moreover, overexpression of the seed-specific transcription factor in transgenic tobacco (*Nicotiana tabacum*) plants, Heat Shock Factor A9, which extends seed longevity by increasing the amount of heat shock proteins (Prieto-Dapena et al., 2006; Kotak et al., 2007). These molecular studies indicated the complex genetic nature of seed longevity.

Genetic loci associated with seed longevity can be identified by exposing seeds to artificial aging conditions. Several seed longevity associated genetic loci therefore have been identified using approach such as in *Arabidopsis* (Bentsink et al., 2000; Tesnier et al., 2002; Clerkx et al., 2004), rice (Miura et al., 2002; Sasaki et al., 2005; Xue et al., 2008; Jiang et al., 2011), lettuce (Schwember and Bradford, 2010), wheat (Landjeva et al., 2010; Arif et al., 2012, 2017), lucerne (Vandecasteele et al., 2011), oilseed rape (Nagel et al., 2011), maize (Han et al., 2014), tobacco (Agacka-Modoch et al., 2015) and barley (Nagel et al., 2016). Seed aging is controlled by several factors and proved to be a multigenic trait and easily influenced by environments (Bentsink et al., 2000; Clerkx et al., 2004; Rajjou et al., 2008; Arif et al., 2012). Therefore, it is difficult to identify the same quantitative trait loci (QTLs) within the same population, regardless of different storage conditions (Schwember and Bradford, 2010; Arif et al., 2012).

Here, a RIL population derived from a cross between ZB and CS was used to search for QTLs of seed vigor-related traits under artificial aging. ZB is an elite Chinese wheat line with over 100 cultivars derived from it (Gao et al., 2015). This study aims to (1) identify stable QTLs for seed vigor-related traits under artificial aging, (2) search for candidate genes at the detected QTL regions, and (3) obtain comprehensive understanding of the mechanism in seed deterioration.

## Materials and Methods

### Plant Materials and Field Trials

Two hundred and forty-six F12 RILs derived from a cross between ZB and CS were used for QTL mapping. This population previously had been analyzed for yield components, plant height and yield-related physiological traits (Gao et al., 2015). Seeds were collected from plant materials grown at Zhengzhou (ZZ) and Zhoukou (ZK) of Henan province in 2014 (ZZ2014, ZK2014), at Zhengzhou in 2015 (ZZ2015), and at Beijing in 2016 (BJ2016).

The field trials using samples with two or three replicates were designed in randomized complete blocks to avoid environmental effects. Fifty plants were grown in a 1.5 m row in the plots consisted of four rows with a distance of 20 cm in between. Field conditions were maintained according to the local practices.

### Controlled Deterioration Test

Three months after harvest, the wheat seeds were taken to be artificially aged to avoid the effect of dormancy on germination process. The CDT was performed using a closed container with saturated KCl solution to reach 82% relative humidity. Seeds were equilibrated for 4 days at 25°C in the dark. Thereafter the seeds were artificially aged for 2 days at 82% relative humidity and at 44.5°C ± 0.1°C in the dark. Seeds were dried at room temperature for 1 day before the germination test (Bentsink et al., 2000; Rajjou et al., 2008; Landjeva et al., 2010; Zhang et al., 2016).

### Germination Test

The germination process of seeds was done by observing seed vigor-related traits under artificial aging. Test was carried out in Petri dishes containing two pieces of moist filter paper at 23°C under an 18-h light/6-h dark photoperiod. Observations were made every 24 h for 7 days from seed imbibition. Germination was evaluated visually by protrusion of the radicle, with the shortest protruding as the same length of the grain. All germination tests were performed in a fully randomized setup of 50 seeds each line. Germination time, rate and synchronicity were measured during the dynamic germination process of the RIL population. MGT, MGR, GI, GR, Z index, and FCGR were calculated with the obtained data using the following formulas (Primack, 1980; Walker-Simmons, 1988; Ranal and de Santana, 2006; Landjeva et al., 2010):

$$\begin{array}{c} MGT=\overset{7}{\underset{i=1}{\Sigma }}N_{i}t_{i}/\overset{7}{\underset{i=1}{\Sigma }}N_{i},MGR=1/MGT,\\ FCGR=(N_{1}+N_{2}+N_{3})/(total\,grains)\times 100\%,\\ GI=(7\times N_{1}+6\times N_{1}+...+1\times N_{7})/(7\times total\,grains)\times 100\%,\\ GR=(N_{1}+N_{2}+...+N_{7})/(total\,grains)\times 100\%,\\ Z=\Sigma C_{Ni,2}/N,C_{Ni,2}=N_{i}(N_{i}-1)/2,\\ N=\Sigma N_{i}(\Sigma N_{i}-1)/2 \end{array}$$

*N_i_* (the number of seeds germinated in time *i*)

*t_i_* (time from the start of the experiment to the *i*th day)

*C_Ni_*_,2_ (combination of the seeds germinated in the time *i*)

### Statistical Analysis

Analysis of variance (ANOVA) was performed to assess genetic variance among the RILs for seed vigor-related traits under artificial aging. Phenotypic correlation analysis among traits related to seed vigor and the TGW was carried out by the statistical software package SPSS 16.0 (APSS Inc., Chicago, IL, United States) using the average values of each trait.

### QTL Analysis and Identification of Candidate Genes

A total of 246 RILs and their parents were genotyped with the wheat 90K iSelect SNP array (81,587 gene-associated SNPs) (Wang et al., 2014; Gao et al., 2015). QTL analysis was analyzed by IciMapping 4.0 software for seed vigor-related traits after artificial aging treatment, with inclusive composite interval mapping (ICIM) (Li et al., 2007)^1^. Phenotypic values of all lines in each environment and the average of the four environments were used for QTL detection. The walking speed for all QTL detections was chosen at 1.0 centimorgans (cM), with *P* = 0.001 in stepwise regression. Based on 2,000 permutations at a probability of 0.01, the LOD threshold of 2.0 was used for declaring putative QTL. Each QTL overlapping within a 20 cM interval were considered possibly common (Lu et al., 2009). The phenotypic variance explained (PVE) was estimated through stepwise regression (Li et al., 2007; Gao et al., 2015; Liu et al., 2016).

To investigate genes involved in germination process after artificial aging, associated genes were BLAST against the database of *Arabidopsis* in NCBI, with wheat and other relative species, such as *Brachypodium distachyon*, rice, maize and barley^2^. BLAST hits were filtered to an *e*-value threshold of 10^-5^ with an identity higher than 75% (Jin et al., 2016; Liu et al., 2016).

## Results

### Phenotypic Evaluation

In the CDT treatment, two parental lines germinated slightly earlier with a moderate germination rate compared to the means of RILs (**Table 1**). CS germinated earlier and more quickly and showed higher GR, GI, and FCGR than ZB after artificial aging treatment, indicating better storability. Consistently, Landjeva et al. (2010) also found CS with higher seed vigor than the other parental line (“Synthetic”) under artificial aging conditions. The frequency distributions of the six parameters measuring the dynamic germination process were fitted to the normal distribution (Supplementary Figure S1). The trend of these traits mostly exhibited bidirectional heterobeltiosis.

**Table 1:** Phenotypic data analysis in parental and recombinant inbred lines (RILs).

Trait	CS	ZB	RILs			
			
			Mean	SD	Min	Max
MGT	2.62	2.74	2.81	0.14	2.48	3.29
MGR	0.38	0.37	0.36	0.02	0.31	0.41
GI	66.45	45.05	56.59	6.04	34.65	73.04
GR	86.29	59.67	76.20	7.07	48.50	93.50
Z	0.45	0.41	0.41	0.05	0.29	0.55
FCGR	74.29	50.67	60.24	7.61	36.50	83.50


*CS, the paternal cultivar Chinese Spring; ZB, the maternal cultivar Zhou 8425B; Mean, the mean phenotypic data of RILs; SD, standard deviation; Min, the minimum phenotypic data of RILs; Max, the maximum phenotypic data of RILs; MGR, mean germination rate; MGT, mean germination time; GI, weighted germination index; GR, germination ratio; Z, synchrony index; FCGR, first count germination ratio.*

### Correlation Analysis

Correlation analysis was performed to identify putative relation between seed vigor-related traits under artificial aging and TGW (**Table 2**). The data showed TGW was positively correlated with MGR, GI, FCGR and especially with Z significantly, while negatively correlated with MGT. This indicates that the heavier the seed, the more uniform and quicker the germination. The correlation analysis (**Table 2**) showed that MGR and MGT were negatively correlated, with the correlation coefficient -0.989, while, GI, GR, and FCGR were mostly positively correlated with each other with the lowest correlation coefficient 0.917. For Z, it was more related with MGR (0.841) and MGT (-0.811) than others.

**Table 2:** Phenotypic correlations among traits related to seed vigor and TGW.

Traits	MGT	MGR	GI	GR	Z	FCGR	TGW
MGT							
MGR	-0.989^∗∗^						
GI	-0.636^∗∗^	0.652^∗∗^					
GR	-0.453^∗∗^	0.473^∗∗^	0.974^∗∗^				
Z	-0.811^∗∗^	0.841^∗∗^	0.503^∗∗^	0.347^∗∗^			
FCGR	-0.733^∗∗^	0.747^∗∗^	0.977^∗∗^	0.917^∗∗^	0.587^∗∗^		
TGW	-0.126^∗^	0.125	0.025	-0.009	0.131^∗^	0.051	


*^∗^Significant at *P* < 0.05, ^∗∗^Significant at *P* < 0.01. MGT, mean germination time; MGR, mean germination rate; GI, weighted germination index; GR, germination ratio; Z, the synchrony index; FCGR, first count germination ratio; TGW, thousand-grain weight.*

### QTL Analysis of Seed Vigor Related Traits

#### Mean Germination Time

**FIGURE 1:**
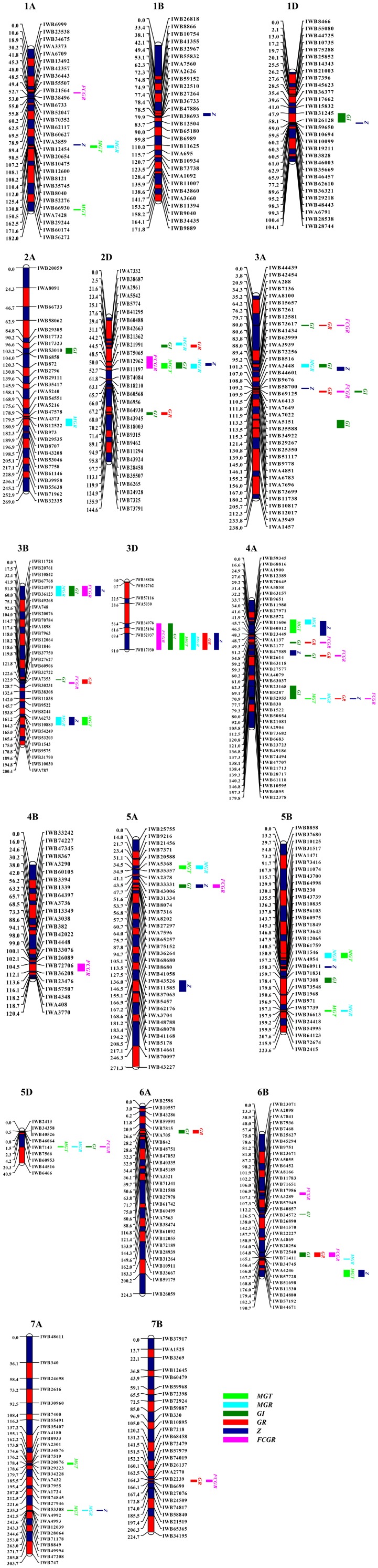
Genetic maps of chromosomes showing QTLs of seed vigor-related traits of MGT, MGR, GI, GR, Z, and FCGR under the germination tests of CDT. MGT, mean germination time; MGR, mean germination rate; GI, weighted germination index; GR, germination ratio; Z, the synchrony index; FCGR, first count germination ratio.

Fourteen QTLs for MGT were identified on chromosomes 1AL (2), 2DS, 3BL, 3DL, 4AS, 4AL, 5AS, 5BL (2), 5DS, 6BL, and 7AL (2) (Supplementary Table S1 and **Figure 1**), designated as *QaMGT.cas-1AL.2*, *QaMGT.cas-1AL.3*, *QaMGT.cas-2DS.2*, *QaMGT.cas-3BL.2*, *QaMGT.cas-3DL*, *QaMGT.cas-4AS*, *QaMGT.cas-4AL.3*, *QaMGT.cas-5AS.1*, *QaMGT.cas-5BL.1*, *QaMGT.cas-5BL.4*, *QaMGT.cas-5DS*, *QaMGT.cas-6BL.2*, *QaMGT.cas-7AL.1* and *QaMGT.cas-7AL.2*, respectively; among these two stable QTLs, *QaMGT.cas-2DS.2* and *QaMGT.cas-4AS* were located in the marker intervals of *IWB21991*∼*IWB11197* and *IWB12389*∼*IWB11606*, explaining 5.4–10.8% and 8.1–9.2% of the phenotypic variance, respectively. Alleles increasing MGT at the *QaMGT.cas-2DS.2* and *QaMGT.cas-4AS* loci came from CS.

#### Mean Germination Rate

MGR is an estimation of germination speed. Sixteen QTLs for MGR were detected on chromosomes 1AL, 2AL, 2DS (2), 3AS, 3BS, 3BL, 3DL, 4AS, 4AL, 5AS, 5BL (2), 5DS, 6BL, and 7AL (Supplementary Table S1 and **Figure 1**), designated as *QaMGR.cas-1AL.2*, *QaMGR.cas-2AL*, *QaMGR.cas-2DS.1*, *QaMGR.cas-2DS.2*, *QaMGR.cas-3AS.2*, *QaMGR.cas-3BS*, *QaMGR.cas-3BL.2*, *QaMGR.cas-3DL*, *QaMGR.cas-4AS*, *QaMGR.cas-4AL.3*, *QaMGR.cas-5AS.1*, *QaMGR.cas-5BL.1*, *QaMGR.cas-5BL.4*, *QaMGR.cas-5DS*, *QaMGR.cas-6BL.2*, and *QaMGR.cas-7AL.2*, respectively; among these, two stable QTLs, *QaMGR.cas-2DS.2* and *QaMGR.cas-4AS* were located in the marker intervals of *IWB21991*∼*IWB11197* and *IWB12389*∼*IWB9651*, explaining 4.6–6.5% and 7.6–10.7% of the phenotypic variance, respectively, and the additive effect was for ZB allele. Interestingly, the two stable QTLs identified for MGR were located on the same region as MGT.

#### Weighted Germination Index

The GI gives the maximum weight to seeds that germinate first and less weight to those that germinate subsequently (Walker-Simmons, 1988). Twenty-one QTLs for GI were identified on chromosomes 1DS, 2AS, 2DS (2), 2DL, 3AS (3), 3AL, 3BS, 3BL, 3DL, 4AL (3), 5AS, 5BL, 5DS, 6AS, and 6BL (2) (Supplementary Table S1 and **Figure 1**), designated as *QaGI.cas-1DS*, *QaGI.cas-2AS*, *QaGI.cas-2DS.1*, *QaGI.cas-2DS.2*, *QaGI.cas-2DL*, *QaGI.cas-3AS.1*, *QaGI.cas-3AS.2*, *QaGI.cas-3AS.3*, *QaGI.cas-3AL*, *QaGI.cas-3BS*, *QaGI.cas-3BL.1*, *QaGI.cas-3DL*, *QaGI.cas-4AL.1*, *QaGI.cas-4AL.2*, *QaGI.cas-4AL.3*, *QaGI.cas-5AS.2*, *QaGI.cas-5BL.3*, *QaGI.cas-5DS*, *QaGI.cas-6AS*, *QaGI.cas-6BL.1*, and *QaGI.cas-6BL.2*, respectively; among these, one stable QTL, *QaGI.cas-3DL* was located in the marker interval of *IWB34976*∼*IWB17930*, accounting for 3.1–6.6% of the phenotypic variance, and increasing GI at *QaGI.cas-3DL* locus was derived from ZB. QTL distribution indicated that both the A and D genomes might be closely related with GI.

#### Germination Ratio

Germination ratio means the measurement of germination capacity, which is measured by the final GR (Landjeva et al., 2010). Twelve QTLs for GR were mapped on chromosomes 2DS, 2DL, 3AS (2), 3BL, 3DL, 4AL (3), 6AS, 6BL, and 7BL (Supplementary Table S1 and **Figure 1**), designated as *QaGR.cas-2DS.2*, *QaGR.cas-2DL*, *QaGR.cas-3AS.1*, *QaGR.cas-3AS.3*, *QaGR.cas-3BL.1*, *QaGR.cas-3DL*, *QaGR.cas-4AL.1*, *QaGR.cas-4AL.2*, *QaGR.cas-4AL.3*, *QaGR.cas-6AS*, *QaGR.cas-6BL.2*, and *QaGR.cas-7BL*, respectively; among these two stable, QTLs *QaGR.cas-3DL* and *QaGR.cas-6BL.2* in the marker intervals of *IWB52937*∼*IWB17930* and *IWA4869∼IWB28256* explained 4.7–5.7% and 3.9–7.2% of the phenotypic variance, respectively. Allele increasing GR was derived from ZB, and for *QaGR.cas-6BL.2* was CS. Meanwhile, *QaGR.cas-3DL* was almost at the same region as *QaGI.cas-3DL*.

#### Synchrony Index

Z index is the synchronization index which measures the synchrony of one seed with another included in the same replication of one treatment (Ranal and de Santana, 2006). The index Z equal to 1 when the germination of all seeds occurred at the same time and Z equal to 0 when at least two seeds could germinate with one at each time (Primack, 1980; Ranal and de Santana, 2006). Seventeen QTLs for Z index were identified on chromosomes 1AL, 1BL, 1DS, 2DS, 3AS (2), 3BS, 3BL, 3DL, 4AS, 4AL (2), 5AS, 5AL, 5BL, 6BL, and 7AL (Supplementary Table S1 and **Figure 1**), designated as *QaZ.cas-1AL.2*, *QaZ.cas-1BL*, *QaZ.cas-1DS*, *QaZ.cas-2DS.2*, *QaZ.cas-3AS.2*, *QaZ.cas-3AS.3*, *QaZ.cas-3BS*, *QaZ.cas-3BL.2*, *QaZ.cas-3DL*, *QaZ.cas-4AS*, *QaZ.cas-4AL.2*, *QaZ.cas-4AL.3*, *QaZ.cas-5AS.2*, *QaZ.cas-5AL*, *QaZ.cas-5BL.2*, *QaZ.cas-6BL.2*, and *QaZ.cas-7AL.2*, respectively; among these, one stable QTL, *QaZ.cas-4AS* was located in the marker intervals of *IWB70645*∼*IWB2177*, explaining 6.4–11.5% of the phenotypic variance, and increasing Z at this locus was derived from ZB. Further, the stable QTL on chromosome 4A identified for Z was also found at the same region as MGT and MGR.

#### First Count Germination Ratio

First count germination ratio is an estimation of germination energy measured as the first count of GR based on the accumulated number of germinated seeds at 3 days after imbibition (Landjeva et al., 2010). Sixteen QTLs for FCGR were found on chromosomes 1AL, 2DS, 3AS (2), 3BS, 3BL, 3DL, 4AL (3), 4B, 5AS, 5DS, 6BS, 6BL, and 7BL (Supplementary Table S1 and **Figure 1**), designated as *QaFCGR.cas-1AL.1*, *QaFCGR.cas-2DS.2*, *QaFCGR.cas-3AS.1*, *QaFCGR.cas-3AS.3*, *QaFCGR.cas-3BS*, *QaFCGR.cas-3BL.1*, *QaFCGR.cas-3DL*, *QaFCGR.cas-4AL.1*, *QaFCGR.cas-4AL.2*, *QaFCGR.cas-4AL.3*, *QaFCGR.cas-4B*, *QaFCGR.cas-5AS.2*, *QaFCGR.cas-5DS*, *QaFCGR.cas-6BS*, *QaFCGR.cas-6BL.2*, and *QaFCGR.cas-7BL*, respectively; among these, two stable QTLs, *QaFCGR.cas-2DS.2* and *QaFCGR.cas-3DL* located in the marker intervals of *IWB42663*∼*IWB11197* and *IWA5030*∼*IWB17930* explained 4.0–13.6% and 3.6–8.3% of the phenotypic variance, respectively, and the additive effect was for ZB allele. In addition, *QaFCGR.cas-2DS.2*, *QaMGT.cas-2DS.2*, and *QaMGR.cas-2DS.2* were found located at the same region on chromosome 2D. Moreover, the stable QTL on chromosome 3D identified for FCGR was the same region as GI.

#### Co-localization of QTLs and Stable Loci for Seed Vigor-Related Traits

Co-localization of QTLs or QTL clusters in this study were detected with each for more than three traits, and 17 QTL clusters associated with seed vigor-related traits after artificial aging were observed on chromosomes 1AL, 2DS, 3AS (3), 3BS, 3BL (2), 3DL, 4AS, 4AL (3), 5AS, 5DS, 6BL, and 7AL (Supplementary Table S1 and **Figure 1**).

In total, 96 QTLs were identified for six germination parameters using the 90K SNP assay linkage map (**Figure 1**). Meanwhile, 10 stable QTLs *QaMGT.cas-2DS.2*, *QaMGR.cas-2DS.2*, *QaFCGR.cas-2DS.2*, *QaGI.cas-3DL*, *QaGR.cas-3DL*, *QaFCGR.cas-3DL*, *QaMGT.cas-4AS*, *QaMGR.cas-4AS*, *QaZ.cas-4AS*, and *QaGR.cas-6BL.2* were detected on chromosomes 2D, 3D, 4A, and 6B across three or four environments (Supplementary Table S1). Further, the analysis of the co-location of QTL clusters demonstrated that 2D, 3D, 4A, and 6B were important chromosomes that related with all the six germination parameters.

## Discussion

Seed aging (or seed deterioration) is an typical quantitative trait which is easily influenced by environments (Bentsink et al., 2000; Rajjou et al., 2008; Ahmed et al., 2016), and germination process is physiologically complex (Bewley and Black, 1994). These make the measurement of wheat seeds germination process after artificial aging much more complicated. The six parameters mentioned above represented different aspects of the dynamic germination process (Primack, 1980; Walker-Simmons, 1988; Ranal and de Santana, 2006; Landjeva et al., 2010). Nguyen et al. (2012) tested the final germination percentage and germination rate to expound the negative correlation between seed longevity and seed dormancy using *Arabidopsis* RILs. Landjeva et al. (2010) used parameters of MGT, MGR, CVt, FCGR, and GR to investigate the germination progress after accelerated aging in wheat D genome introgression lines. Arif et al. (2012) analyzed traits associated with seed longevity by measuring the germination percentage using wheat RILs. Han et al. (2014) used the germination percentage and FCGR for QTL analysis of seed vigor-related traits under artificial aging conditions in maize.

In the present study, we firstly utilized six germination parameters (MGT, MGR, GI, GR, Z, and FCGR) for QTL mapping in wheat. Therefore, the measurement of the dynamic germination progress was more precise, and a total of 96 QTLs were identified for six germination parameters using the 90K SNP assay linkage map (**Figure 1**). QTL clusters were detected in the region for more than three traits, and 17 QTL-rich regions were found (**Figure 1**). Across three or four environments, 10 stable QTLs were detected on chromosomes 2D, 3D, 4A, and 6B (Supplementary Table S1). The correlation analysis (**Table 2**) showed that MGR and MGT were correlated, while, GI, GR, and FCGR were mostly related with each other. For Z, it was more related with MGR and MGT than others. Interestingly, the co-location of some traits was consistent with the result of correlation analysis (**Table 2** and **Figure 1**). MGR, MGT, and Z were mainly co-located on chromosomes 1A, 2D, 3B, 3D, 4A, and 7A, while GI, GR, and FCGR were generally clustered on chromosomes 3A, 3B, 3D, 4A, and 6B (**Figure 1**). These findings may be very helpful to comprehensively understand the regulation of wheat germination during seed aging.

### Comparison With Previous Studies

Genetic loci associated with seed longevity have been identified in different species by QTL analysis. Nagel et al. (2016) found a major seed aging related locus on chromosome 2H in barley, which was co-linear to wheat chromosome 2D (Muñoz-Amatriaín et al., 2015). The locus on chromosome 2H was detected on the end of the long arm, while our QTL on chromosome 2D located near the centromere. Landjeva et al. (2010) found QTLs on chromosomes 1D associated with FCGR and 5D associated with GR, respectively, by measuring the germination after artificial aging using wheat D genome introgression lines, however, these were not appeared in our study. Arif et al. (2012) identified QTLs on chromosomes 1AL, 1DS, 1DL (2), 2AS, 2DL, 3BS, 3DL, 6BS, and 7AS in wheat were associated with seed longevity by measuring the germination percentage. Coincidently a stable QTL *QaGR.cas-3DL* found in this study on the chromosome 3DL flanked by *IWB52937* and *IWB17930*, is in the same chromosome bin (3DL2-0.27-0.81) as *QLng.ipk-3D* (Arif et al., 2012). Therefore, *QaGR.cas-3DL* is likely to be the same QTL as *QLng.ipk-3D*. However, other QTLs were not detected, which could be attributed to different genetic materials used. Owing to the poor research on wheat longevity related traits, expedition of systematic studies on QTLs associated with wheat longevity may overcome this issue. QTLs identified in this study (except *QaGR.cas-3DL*) for seed vigor-related traits under artificial aging were likely to be new.

### Candidate Genes Identified by QTL Analysis

A total of 96 QTLs were detected in the study with three major stable QTL clusters, which were considered as important regions in association with seed storability. Therefore, the candidate genes of the three regions were BLAST against the database of *Arabidopsis* and some cereal genome sequences (Supplementary Table S2).

One of the stable QTL-rich regions for seed storability on chromosome 2D was detected in our study, flanked by *IWB21991* and *IWB11197* from 46 to 51 cM. QTL on chromosome 2D had a strong effect on wheat deterioration by measuring the process of germination after artificial aging treatment. By comparison, locus on chromosome 2D was not detected in the control assay without aging treatment. So we deem that this QTL was an important pleiotropic locus for regulating seed storage and seed germination. The region from 46 to 51 cM on chromosome 2D contained 4 SNP markers: *IWB21991*, *IWB75065*, *IWB12962*, and *IWB11197*. Both *IWB21991* and *IWB12962* were associated with the starch synthase family gene (*SSIIIb* gene) which functions in starch synthesizing and binding. Basavarajappa et al. (1991) and Han et al. (2014) suggested that seed aging affected carbohydrate metabolism in maize, moreover, Zhang et al. (2016) analyzed the proteomic of artificially aged rice seeds and found that the abundance of sugar metabolism proteins had a great change. These imply that carbohydrate metabolism plays a complicated and important role in seed aging. Nagel et al. (2016) found an important QTL on chromosome 2H, which showed co-linearity to QTLs detected for rice seed deterioration (Sasaki et al., 2005, 2015; Stein et al., 2007), might be associated with seed vigor of aged barley seed. The region contained a gene, annotated as encoding trehalose-6-phosphate phosphatase (Sasaki et al., 2005). Particularly, the database of KEGG biological pathways showed that both trehalose-6-phosphate phosphatase and soluble starch synthase belonged to starch and sucrose metabolism. Therefore, we speculate that the QTL locus on wheat chromosome 2D might be involved in starch and sucrose metabolism to regulate seed deterioration. On the other hand, *TaSdr* genes associated with tolerance to pre-harvest sprouting in common wheat were cloned, and *TaSdr-A1* and *TaSdr-B1* were mapped on chromosomes 2A and 2B (Sugimoto et al., 2010; Zhang et al., 2014, 2017). Owing to the extremely high sequence similarity between *TaSdr-A1*, *TaSdr-B1*, and *TaSdr-D1* (Zhang et al., 2014), *TaSdr-D1* is very likely to be located on chromosome 2D. As from the positive correlation between seed vigor and dormancy, we also speculate that *TaSdr-D1* might be a good candidate for the QTL detected on wheat chromosome 2D. However, additional experimental analyses are needed to confirm this speculation.

Meanwhile, another QTL on the long arm of chromosome 3D identified in our study was also detected by Arif et al. (2012). Seed aging is a multigenic trait and easily influenced by environments, which made it difficult to identify the same QTL even using the same population (Schwember and Bradford, 2010; Arif et al., 2012). Thus, we speculate that this locus is an important region associated with seed storability. Several candidate genes in this region were also mainly associated with carbohydrate metabolism (Supplementary Table S2). Moreover, a stable QTL on chromosome 4A was detected to be a pleiotropic locus affecting MGT, MGR, and Z. The sequences of SNP markers tightly linked to seed vigor-related traits after artificial aging corresponded to stress related genes (Supplementary Table S2), which may play a role in surviving the severe conditions. The candidate gene analysis results indicate that a complex gene network may be involved in the regulation of seed longevity in wheat.

## Conclusion

In this study, 96 QTLs was detected for six germination parameters associated with seed deterioration using a RIL population derived from the cross between ZB and CS. Co-localization of QTLs or QTL clusters in a region for more than three parameters were identified, and 17 QTL-rich regions, were, respectively, found on chromosomes 1AL, 2DS, 3AS (3), 3BS, 3BL (2), 3DL, 4AS, 4AL (3), 5AS, 5DS, 6BL, and 7AL, exhibiting pleiotropic effects. Ten stable QTLs were identified across three or four environments on chromosomes 2D, 3D, 4A, and 6B (*QMGT.cas-2DS.2*, *QMGR.cas-2DS.2*, *QFCGR.cas-2DS.2*, *QGI.cas-3DL*, *QGR.cas-3DL*, *QFCGR.cas-3DL*, *QMGT.cas-4AS*, *QMGR.cas-4AS*, *QZ.cas-4AS*, and *QGR.cas-6BL.2*). Interestingly, our QTL mapping analysis identified a more stable QTL-rich region for seed storability on chromosome 2D, containing candidate genes encoding soluble starch synthase. However, whether the soluble starch synthase regulating seed aging remains to be investigated in future. In summary, 2DS, 3DL, 4AS, and 6BL are considered as important regions in association with seed storability. Meanwhile, the newly identified QTLs and SNP markers may provide valuable information and could act as useful targets for marker-assisted selection in wheat breeding.

## Author Contributions

YL designed the experiment. JZ carried out the experiment and wrote the paper. JL helped with the data analysis. FG, GY, HC, ZW, FC, XL, JX, TC, LL, and YL participated in field trials. HC and XX assisted in English edition of the manuscript. All authors have read and approved this manuscript.

## Conflict of Interest Statement

The authors declare that the research was conducted in the absence of any commercial or financial relationships that could be construed as a potential conflict of interest.

## Footnotes

1 http://www.isbreeding.net/software/?type=detail&id=18

2 http://www.ncbi.nlm.nih.gov/

## Abbreviations

CDT: controlled deterioration test
CS: Chinese Spring
FCGR: first count germination ratio
GI: weighted germination index
GR: germination ratio
LOD: logarithm of odds
MGR: mean germination rate
MGT: mean germination time
QTLs: quantitative trait loci
RIL: recombinant inbred line
SNP: single nucleotide polymorphism
TGW: thousand-grain weight
Z: the synchrony index
ZB: Zhou 8425B

## References

[B1] Agacka-Modoch M. Nagel M. Doroszewska T. Lewis R. S. Börner A. (2015). Mapping quantitative trait loci determining seed longevity in tobacco (*Nicotiana tabacum* L.). *Euphytica* 202 479–486. 10.1007/s10681-015-1355-x

[B2] Ahmed Z. Yang H. Fu Y. B. (2016). The associative changes in scutellum nuclear content and morphology with viability loss of naturally aged and accelerated aging wheat (*Triticum aestivum*) seeds. *Front. Plant Sci.* 7:1474. 10.3389/fpls.2016.01474 27729925 PMC5037135

[B3] Arif M. A. Nagel M. Lohwasser U. Börner A. (2017). Genetic architecture of seed longevity in bread wheat (*Triticum aestivum* L.). *J. Biosci.* 42 81–89. 10.1007/s12038-016-9661-6 28229967

[B4] Arif M. A. Nagel M. Neumann K. Kobiljski B. Lohwasser U. Börner A. (2012). Genetic studies of seed longevity in hexaploid wheat using segregation and association mapping approaches. *Euphytica* 186 1–13. 10.1007/s10681-011-0471-5

[B5] Bailly C. (2004). Active oxygen species and antioxidants in seed biology. *Seed Sci. Res.* 14 93–107. 10.1079/SSR2004159

[B6] Basavarajappa B. S. Shekar Shetty H. Prakash H. S. (1991). Membrane deterioration and other biochemical changes, associated with accelerated aging of maize seeds. *Seed Sci. Technol.* 19 279–286.

[B7] Bentsink L. Alonso-Blanco C. Vreugdenhil D. Tesnier K. Groot S. P. C. Koornneef M. (2000). Genetic analysis of seed-soluble oligosaccharides in relation to seed storability of *Arabidopsis*. *Plant Physiol.* 124 1595–1604. 10.1104/pp.124.4.1595 11115877 PMC59858

[B8] Bentsink L. Jowett J. Hanhart C. J. Koornneef M. (2006). Cloning of DOG1, a quantitative trait locus controlling seed dormancy in *Arabidopsis*. *Proc. Natl. Acad. Sci. U.S.A.* 103 17042–17047. 10.1073/pnas.0607877103 17065317 PMC1636575

[B9] Bewley J. D. Black M. (1994). *Seeds: Physiology of Development and Germination.* New York, NY: Plenum Press 10.1007/978-1-4899-1002-8

[B10] Bewley J. D. Bradford K. J. Hilhorst H. W. M. Nonogaki H. (2013). *Seeds, Physiology of Development, Germination and Dormancy.* New York, NY: Springer.

[B11] Borisjuk L. Rolletschek H. (2009). The oxygen status of the developing seed. *New Phytol.* 182 17–30. 10.1111/j.1469-8137.2008.02752.x 19207684

[B12] Bueso E. Muñoz-Bertomeu J. Campos F. Brunaud V. Martínez L. Sayas E. (2014). *Arabidopsis thaliana* HOMEOBOX25 uncovers a role for gibberellins in seed longevity. *Plant Physiol.* 164 999–1010. 10.1104/pp.113.232223 24335333 PMC3912122

[B13] Cantliffe D. J. Shuler K. D. Guedes A. C. (1981). Overcoming seed thermodormancy in a heat-sensitive romaine lettuce by seed priming. *Hortscience* 16 196–198.

[B14] Clerkx E. J. M. Koornneef M. (2003). Characterization of green seed, an enhancer of abi3-1 in *Arabidopsis* that affects seed longevity. *Plant Physiol.* 132 1077–1084. 10.1104/pp.103.022715 12805635 PMC167045

[B15] Clerkx E. J. M. Vries B. D. Ruys G. J. Groot S. P. C. Koornneef M. (2004). Genetic differences in seed longevity of various *Arabidopsis* mutants. *Physiol. Plant.* 121 448–461. 10.1111/j.1399-3054.2004.00339.x

[B16] Corbineau F. Gay-Mathieu C. Vinel D. Côme D. (2002). Decrease in sunflower (*Helianthus annuus*) seed viability caused by high temperature as related to energy metabolism, membrane damage and lipid composition. *Physiol. Plant.* 116 489–496. 10.1034/j.1399-3054.2002.1160407.x

[B17] Delouche J. C. Baskin C. C. (1973). Accelerated ageing techniques for predicting the relative storability of seed lots. *Seed Sci. Technol.* 1 427–452.

[B18] Gao F. M. Wen W. E. Liu J. D. Rasheed A. Yin G. H. Xia X. C. (2015). Genome-wide linkage mapping of QTL for yield components, plant height and yield-related physiological traits in the Chinese wheat cross Zhou 8425B/Chinese Spring. *Front. Plant Sci.* 6:1099. 10.3389/fpls.2015.01099 26734019 PMC4683206

[B19] Han Z. P. Ku L. X. Zhang Z. Z. Zhang J. Guo S. L. Liu H. Y. (2014). QTLs for seed vigor-related traits identified in maize seeds germinated under artificial aging conditions. *PLoS One* 9:e92535. 10.1371/journal.pone.0092535 24651614 PMC3961396

[B20] Hendry G. A. F. (1993). Oxygen, free radical processes and seed longevity. *Seed Sci. Res.* 3 141–153. 10.1017/S0960258500001720

[B21] Jiang W. Lee J. Jin Y. M. Qiao Y. Piao R. Jang S. M. (2011). Identification of QTLs for seed germination capability after various storage periods using two RIL populations in rice. *Mol. Cells* 31 385–392. 10.1007/s10059-011-0049-z 21399992 PMC3933968

[B22] Jin H. Wen W. E. Liu J. D. Zhai S. N. Zhang Y. Yan J. (2016). Genome-wide QTL mapping for wheat processing quality parameters in a Gaocheng 8901/Zhoumai 16 recombinant inbred line population. *Front. Plant Sci.* 7:1032. 10.3389/fpls.2016.01032 27486464 PMC4949415

[B23] Kotak S. Larkindale J. Lee U. Koskull-Döring P. V. Vierling E. Scharf K. D. (2007). Complexity of the heat stress response in plants. *Curr. Opin. Plant Biol.* 10 310–316. 10.1016/j.pbi.2007.04.011 17482504

[B24] Landjeva S. Lohwasser U. Börner A. (2010). Genetic mapping within the wheat D genome reveals QTL for germination, seed vigour and longevity, and early seedling growth. *Euphytica* 171 129–143. 10.1007/s10681-009-0016-3

[B25] Li H. H. Ye G. Y. Wang J. K. (2007). A modified algorithm for the improvement of composite interval mapping. *Genetics* 175 361–374. 10.1534/genetics.106.06681 17110476 PMC1775001

[B26] Liu J. D. He Z. H. Wu L. Bai B. Wen W. E. Xie C. (2016). Genome-wide linkage mapping of QTL for black point reaction in bread wheat (*Triticum aestivum* L.). *Theor. Appl. Genet.* 129 2179–2190. 10.1007/s00122-016-2766-3 27531362

[B27] Lu Y. M. Lan C. X. Liang S. S. Zhou X. C. Liu D. Zhou G. (2009). QTL mapping for adult-plant resistance to stripe rust in Italian common wheat culativars Libellula and Strampelli. *Theor. Appl. Genet.* 119 1349–1359. 10.1007/s00122-009-1139-6 19756474

[B28] McDonald M. B. (1999). Seed deterioration: physiology, repair and assessment. *Seed Sci. Technol.* 27 177–237.

[B29] Miura K. Lin S. Y. Yano M. Nagamine T. (2002). Mapping quantitative trait loci controlling seed longevity in rice (*Oryza sativa* L.). *Theor. Appl. Genet.* 104 981–986. 10.1007/s00122-002-0872-x 12582603

[B30] Muñoz-Amatriaín M. Lonardi S. Luo M. Madishetty K. Svensson J. T. Moscou M. J. (2015). Sequencing of 15 622 gene-bearing BACs clarifies the gene-dense regions of the barley genome. *Plant J.* 84 216–227. 10.1111/tpj.12959 26252423 PMC5014227

[B31] Nagel M. Kodde J. Pistrick S. Mascher M. Börner A. Groot S. P. (2016). Barley seed aging: genetics behind the dry elevated pressure of oxygen aging and moist controlled deterioration. *Front. Plant Sci.* 7:388. 10.3389/fpls.2016.00388 27066038 PMC4814755

[B32] Nagel M. Rosenhauer M. Willner E. Snowdon R. J. Friedt W. Börner A. (2011). Seed longevity in oilseed rape (*Brassica napus* L.) genetic variation and QTL mapping. *Plant Genet. Resour.* 9 260–263. 10.1017/S1479262111000372

[B33] Nguyen T. P. Keizer P. van Eeuwijk F. Smeekens S. Bentsink L. (2012). Natural variation for seed longevity and seed dormancy are negatively correlated in *Arabidopsis*. *Plant Physiol.* 160 2083–2092. 10.1104/pp.112.206649 23085841 PMC3510133

[B34] Priestley D. A. (1986). *Seed Aging.* New York, NY: Cornell University Press.

[B35] Prieto-Dapena P. Castaño R. Almoguera C. Jordano J. (2006). Improved resistance to controlled deterioration in transgenic seeds. *Plant Physiol.* 142 1102–1112. 10.1104/pp.106.087817 16998084 PMC1630740

[B36] Primack R. B. (1980). Variation in the phenology of natural populations of montane shrubs in New Zealand. *J. Ecol.* 68 849–862. 10.2307/2259460

[B37] Rajjou L. Lovigny Y. Groot S. P. C. Belghazi M. Job C. Job D. (2008). Proteome wide characterization of seed aging in *Arabidopsis*: a comparison between artificial and natural aging protocols. *Plant Physiol.* 148 620–641. 10.1104/pp.108.123141 18599647 PMC2528126

[B38] Ranal M. A. de Santana D. G. (2006). How and why to measure the germination process? *Braz. J. Bot.* 29 1–11. 10.1590/S0100-84042006000100002

[B39] Sasaki K. Fukuta Y. Sato T. (2005). Mapping of quantitative trait loci controlling seed longevity of rice (*Oryza sativa* L.) after various periods of seed storage. *Plant Breed.* 124 361–366. 10.1111/j.1439-0523.2005.01109.x

[B40] Sasaki K. Takeuchi Y. Miura K. Yamaguchi T. Ando T. Ebitani T. (2015). Fine mapping of a major quantitative trait locus, qLG-9, that controls seed longevity in rice (*Oryza sativa* L.). *Theor. Appl. Genet.* 128 769–778. 10.1007/s00122-015-2471-7 25687128

[B41] Sattler S. E. Gilliland L. U. Magallaneslundback M. Pollard M. Dellapenna D. (2004). Vitamin E is essential for seed longevity and for preventing lipid peroxidation during germination. *Plant Cell* 16 1419–1432. 10.1105/tpc.021360 15155886 PMC490036

[B42] Schwember A. R. Bradford K. J. (2010). Quantitative trait loci associated with longevity of lettuce seeds conventional under conditions controlled deterioration storage. *J. Exp. Bot.* 61 4423–4436. 10.1093/jxb/erp248 20693410 PMC2955753

[B43] Smith M. T. Berjak P. (1995). “Deteriorative changes associated with the loss of viability of stored desiccation-tolerant and desiccation-sensitive seeds,” in *Seed Development and Germination*, ed. Galili G. (New York, NY: Marcel Dekker), 701–746.

[B44] Smith R. D. Dickie J. D. Linington S. H. Pritchard H. W. Probert R. J. (2003). *Seed Conservation: Turning Science into Practice.* Chicago, IL: University of Chicago Press.

[B45] Stein N. Prasad M. Scholz U. Thiel T. Zhang H. N. Wolf M. (2007). A 1,000-loci transcript map of the barley genome: new anchoring points for integrative grass genomics. *Theor. Appl. Genet.* 114 823–839. 10.1007/s00122-006-0480-2 17219208

[B46] Sugimoto K. Takeuchi Y. Ebana K. Miyao A. Hirochika H. Hara N. (2010). Molecular cloning of Sdr4, a regulator involved in seed dormancy and domestication of rice. *Proc. Natl. Acad. Sci. U.S.A.* 107 5792–5797. 10.1073/pnas.0911965107 20220098 PMC2851884

[B47] Tesnier K. Strookman-Donkers H. M. Van Pijlen J. G. Van der Geest A. H. M. Bino R. J. Groot S. P. C. (2002). A controlled deterioration test for *Arabidopsis thaliana* reveals genetic variation in seed quality. *Seed Sci. Technol.* 30 149–165.

[B48] Vandecasteele C. Teulat-Merah B. Morere-Le Paven M. C. Leprince O. Ly Vu B. (2011). Quantitative trait loci analysis reveals a correlation between the ratio of sucrose/raffinose family oligosaccharides and seed vigour in *Medicago truncatula*. *Plant Cell Environ.* 34 1473–1487. 10.1111/j.1365-3040.2011.02346.x 21554325

[B49] Walker-Simmons M. (1988). Enhancement of ABA responsiveness in wheat embryos by high temperature. *Plant Cell Environ.* 11 769–775. 10.1111/j.1365-3040.1988.tb01161.x

[B50] Wang S. C. Wong D. Forrest K. Allen A. Chao S. M. Huang B. E. (2014). Characterization of polyploid wheat genomic diversity using a high-density 90000 single nucleotide polymorphism array. *Plant Biotechnol. J.* 12 87–96. 10.1111/pbi.12183 24646323 PMC4265271

[B51] Wang Z. Cao H. Sun Y. Z. Li X. Y. Chen F. Y. Carles A. (2013). *Arabidopsis* paired amphipathic helix proteins SNL1 and SNL2 redundantly regulate primary seed dormancy via abscisic acid-ethylene antagonism mediated by histone deacetylation. *Plant Cell* 25 149–166. 10.1105/tpc.112.108191 23371947 PMC3584531

[B52] Waterworth W. M. Footitt S. Bray C. M. Finch-Savage W. E. West C. E. (2016). DNA damage checkpoint kinase ATM regulates germination and maintains genome stability in seeds. *Proc. Natl. Acad. Sci. U.S.A.* 113 9647–9652. 10.1073/pnas.1608829113 27503884 PMC5003248

[B53] Xue Y. Zhang S. Yao Q. Peng R. Xiong A. Li X. (2008). Identification of quantitative trait loci for seed storability in rice (*Oryza sativa* L.). *Euphytica* 164 739–744. 10.1007/s10681-008-9696-3

[B54] Zhang Y. J. Miao X. L. Xia X. C. He Z. H. (2014). Cloning of seed dormancy genes (TaSdr) associated with tolerance to pre-harvest sprouting in common wheat and development of a functional marker. *Theor. Appl. Genet.* 127 855–866. 10.1007/s00122-014-2262-6 24452439

[B55] Zhang Y. J. Xia X. C. He Z. H. (2017). The seed dormancy allele TaSdr-A1a associated with pre-harvest sprouting tolerance is mainly present in Chinese wheat landraces. *Theor. Appl. Genet.* 130 81–89. 10.1007/s00122-016-2793-0 27650191

[B56] Zhang Y. X. Xu H. H. Liu S. J. Li N. Wang W. Q. Møller I. M. (2016). Proteomic analysis reveals different involvement of embryo and endosperm proteins during aging of Yliangyou 2 hybrid rice seeds. *Front. Plant Sci.* 7:1394. 10.3389/fpls.2016.01394 27708655 PMC5031166

