# Supplementary material for: Genome-Wide Linkage Mapping Reveals QTLs for Seed Vigor-Related Traits Under Artificial Aging in Common Wheat (Triticum aestivum)
Source: Front Plant Sci. 2018 Jul 27;9:1101. doi: 10.3389/fpls.2018.01101 (PMC6073742; doi:10.3389/fpls.2018.01101)
Supplement: Supplementary file 1 [file Table_1.docx]

**Table S1** QTLs identified for seed vigor-related traits under artificial aging in the Zhou8425B/Chinese Spring population

| **Trait** | **Environment** | **QTL** | **Position** | **Marker interval** | **LOD** | **PVE** | **Add** |
| --- | --- | --- | --- | --- | --- | --- | --- |
| MGT | ZK2014 | *QaMGT.cas-5AS.1* | 33 | *IWB20588~IWA5368* | 2.21 | 9.7 | -0.062 |
|  |  | *QaMGT.cas-6BL.2* | 183 | *IWB57192~IWB44671* | 2.02 | 3.6 | -0.040 |
|  |  | *QaMGT.cas-7AL.1* | 177 | *IWB7519~IWB20876* | 2.80 | 6.4 | -0.052 |
|  |  | *QaMGT.cas-7AL.2* | 243 | *IWA4993~IWB12039* | 2.60 | 4.7 | 0.044 |
|  | ZZ2014 | *QaMGT.cas-1AL.3* | 180 | *IWA4518~IWB8643* | 2.16 | 3.8 | 0.043 |
|  |  | *QaMGT.cas-2DS.2* | 51 | *IWB12962~IWB11197* | 2.30 | 7.0 | 0.058 |
|  |  | *QaMGT.cas-4AS* | 30 | *IWB70645~IWA5858* | 2.64 | 8.9 | 0.065 |
|  |  | *QaMGT.cas-5BL.1* | 135 | *IWB230~IWB43739* | 2.25 | 4.1 | 0.044 |
|  | ZZ2015 | *QaMGT.cas-2DS.2* | 46 | *IWB21991~IWB75065* | 2.54 | 5.4 | 0.062 |
|  |  | *QaMGT.cas-3BL.2* | 166 | *IWB53203~IWB1543* | 2.03 | 3.8 | -0.052 |
|  |  | *QaMGT.cas-5DS* | 20 | *IWB60953~IWB44516* | 2.17 | 3.9 | 0.053 |
|  | BJ2016 | *QaMGT.cas-3DL* | 91 | *IWB52937~IWB17930* | 2.82 | 4.6 | 0.062 |
|  |  | *QaMGT.cas-4AS* | 44 | *IWB3572~IWB11606* | 2.92 | 8.1 | 0.081 |
|  |  | *QaMGT.cas-4AL.3* | 139 | *IWB21713~IWB28717* | 3.02 | 5.2 | 0.065 |
|  | Average | *QaMGT.cas-1AL.2* | 109 | *IWB8121~IWB35745* | 2.66 | 4.2 | -0.029 |
|  |  | *QaMGT.cas-2DS.2* | 51 | *IWB12962~IWB11197* | 4.38 | 10.8 | 0.047 |
|  |  | *QaMGT.cas-4AS* | 28 | *IWB12389~IWB70645* | 5.97 | 9.2 | 0.043 |
|  |  | *QaMGT.cas-5BL.4* | 199 | *IWB36613~IWB24418* | 3.37 | 5.0 | 0.032 |
|  |  | *QaMGT.cas-7AL.2* | 243 | *IWA4993~IWB12039* | 2.89 | 4.6 | 0.031 |
| MGR | ZK2014 | *QaMGR.cas-2DS.2* | 48 | *IWB21991~IWB75065* | 2.65 | 4.6 | -0.006 |
|  |  | *QaMGR.cas-5AS.1* | 33 | *IWB20588~IWA5368* | 4.45 | 19.4 | 0.012 |
|  |  | *QaMGR.cas-6BL.2* | 167 | *IWA4246~IWB57728* | 2.75 | 4.9 | 0.007 |
|  |  | *QaMGR.cas-7AL.2* | 243 | *IWA4993~IWB12039* | 2.31 | 4.0 | -0.006 |
|  | ZZ2014 | *QaMGR.cas-2DS.2* | 51 | *IWB12962~IWB11197* | 2.05 | 6.3 | -0.007 |
|  |  | *QaMGR.cas-3BS* | 12 | *IWB11728~IWA5347* | 2.15 | 3.8 | -0.005 |
|  |  | *QaMGR.cas-4AS* | 30 | *IWB70645~IWA5858* | 2.88 | 10.0 | -0.008 |
|  |  | *QaMGR.cas-5BL.1* | 135 | *IWB230~IWB43739* | 2.10 | 3.8 | -0.005 |
|  | ZZ2015 | *QaMGR.cas-2AL* | 187 | *IWB973~IWB29535* | 2.11 | 5.4 | -0.007 |
|  |  | *QaMGR.cas-2DS.2* | 47 | *IWB21991~IWB75065* | 2.35 | 5.0 | -0.007 |
|  |  | *QaMGR.cas-3BL.2* | 166 | *IWB53203~IWB1543* | 2.03 | 4.0 | 0.006 |
|  |  | *QaMGR.cas-5DS* | 20 | *IWB60953~IWB44516* | 2.07 | 3.9 | -0.006 |
|  | BJ2016 | *QaMGR.cas-2DS.1* | 27 | *IWB5774~IWB41295* | 2.15 | 3.9 | -0.007 |
|  |  | *QaMGR.cas-3DL* | 91 | *IWB52937~IWB17930* | 2.90 | 5.0 | -0.008 |
|  |  | *QaMGR.cas-4AS* | 33 | *IWB63157~IWB9651* | 3.39 | 10.7 | -0.012 |
|  |  | *QaMGR.cas-4AL.3* | 139 | *IWB21713~IWB28717* | 3.25 | 5.8 | -0.009 |
|  | Average | *QaMGR.cas-1AL.2* | 109 | *IWB8121~IWB35745* | 3.79 | 6.5 | 0.005 |
|  |  | *QaMGR.cas-2DS.2* | 51 | *IWB12962~IWB11197* | 2.61 | 6.5 | -0.005 |
|  |  | *QaMGR.cas-3AS.2* | 83 | *IWB41434~IWB63999* | 3.03 | 5.1 | -0.004 |
|  |  | *QaMGR.cas-3DL* | 73 | *IWB52937~IWB17930* | 2.18 | 3.8 | -0.004 |
|  |  | *QaMGR.cas-4AS* | 28 | *IWB12389~IWB70645* | 4.68 | 7.6 | -0.005 |
|  |  | *QaMGR.cas-5BL.4* | 199 | *IWB36613~IWB24418* | 2.04 | 3.1 | -0.003 |
|  |  |  |  |  |  | (Contined) | |
|  |  |  |  |  |  |  | |
| **Trait** | **Environment** | **QTL** | **Position** | **Marker interval** | **LOD** | **PVE** | **Add** |
| GI | ZK2014 | *QaGI.cas-1DS* | 54 | *IWB31245~IWB26128* | 2.00 | 7.3 | -2.711 |
|  |  | *QaGI.cas-2DL* | 97 | *IWB43924~IWB28458* | 2.20 | 5.4 | -2.362 |
|  |  | *QaGI.cas-3AS.3* | 112 | *IWA7022~IWA5151* | 3.61 | 5.6 | 2.377 |
|  |  | *QaGI.cas-3DL* | 57 | *IWB34976~IWB25194* | 2.01 | 3.1 | -1.785 |
|  |  | *QaGI.cas-5BL.3* | 165 | *IWB71831~IWB7308* | 2.16 | 4.3 | 2.088 |
|  |  | *QaGI.cas-6AS* | 32 | *IWB48751~IWB47853* | 2.64 | 4.2 | 2.045 |
|  |  | *QaGI.cas-6BL.1* | 107 | *IWB17986~IWA3289* | 2.01 | 3.0 | 1.724 |
|  |  | *QaGI.cas-6BL.2* | 159 | *IWA4869~IWB28256* | 2.84 | 4.4 | 2.250 |
|  | ZZ2014 | *QaGI.cas-2AS* | 98 | *IWB17323~IWB53010* | 2.01 | 3.6 | -1.736 |
|  |  | *QaGI.cas-4AL.1* | 60 | *IWB27577~IWA4079* | 3.99 | 7.5 | -2.495 |
|  | ZZ2015 | *QaGI.cas-2DS.2* | 45 | *IWB21991~IWB75065* | 2.28 | 4.6 | -1.883 |
|  |  | *QaGI.cas-5DS* | 20 | *IWB60953~IWB44516* | 2.01 | 3.6 | -1.662 |
|  | BJ2016 | *QaGI.cas-3AS.1* | 35 | *IWB7136~IWA8100* | 2.70 | 4.1 | -2.861 |
|  |  | *QaGI.cas-3DL* | 91 | *IWB52937~IWB17930* | 4.39 | 6.6 | -2.754 |
|  |  | *QaGI.cas-4AL.2* | 79 | *IWB830~IWB1522* | 3.81 | 8.0 | -3.261 |
|  |  | *QaGI.cas-4AL.3* | 139 | *IWB21713~IWB28717* | 4.04 | 6.3 | -2.680 |
|  |  | *QaGI.cas-5AS.2* | 55 | *IWB8074~IWB7316* | 2.23 | 4.3 | -2.211 |
|  |  | *QaGI.cas-5BL.3* | 159 | *IWB60911~IWB71831* | 2.04 | 2.9 | -1.840 |
|  | Average | *QaGI.cas-2DS.1* | 28 | *IWB41295~IWB60488* | 3.69 | 8.2 | -1.751 |
|  |  | *QaGI.cas-2DS.2* | 51 | *IWB12962~IWB11197* | 2.24 | 6.1 | -1.497 |
|  |  | *QaGI.cas-3AS.2* | 82 | *IWB41434~IWB63999* | 2.23 | 3.8 | -1.178 |
|  |  | *QaGI.cas-3AS.3* | 112 | *IWA7022~IWA5151* | 2.04 | 3.2 | 1.073 |
|  |  | *QaGI.cas-3AL* | 155 | *IWA4851~IWA6783* | 2.04 | 3.3 | 1.263 |
|  |  | *QaGI.cas-3BS* | 12 | *IWB11728~IWA5347* | 2.35 | 3.7 | -1.217 |
|  |  | *QaGI.cas-3BL.1* | 118 | *IWB7963~IWB12064* | 2.26 | 3.5 | -1.150 |
|  |  | *QaGI.cas-3DL* | 86 | *IWB52937~IWB17930* | 2.75 | 5.6 | -1.427 |
|  |  | *QaGI.cas-4AL.3* | 128 | *IWB23723~IWB49186* | 2.11 | 4.6 | -1.293 |
|  |  | *QaGI.cas-6BL.2* | 159 | *IWA4869~IWB28256* | 4.21 | 7.0 | 1.702 |
| GR | ZK2014 | *QaGR.cas-2DL* | 97 | *IWB43924~IWB28458* | 2.16 | 5.3 | -2.769 |
|  |  | *QaGR.cas-3AS.3* | 112 | *IWA7022~IWA5151* | 3.62 | 5.7 | 2.818 |
|  |  | *QaGR.cas-6AS* | 32 | *IWB48751~IWB47853* | 2.88 | 4.6 | 2.529 |
|  |  | *QaGR.cas-6BL.2* | 159 | *IWA4869~IWB28256* | 2.51 | 3.9 | 2.495 |
|  | ZZ2014 | *QaGR.cas-3DL* | 91 | *IWB52937~IWB17930* | 2.85 | 4.7 | -2.371 |
|  |  | *QaGR.cas-4AL.1* | 60 | *IWB27577~IWA4079* | 3.00 | 5.3 | -2.527 |
|  |  | *QaGR.cas-6BL.2* | 159 | *IWA4869~IWB28256* | 2.52 | 4.1 | 2.378 |
|  |  | *QaGR.cas-7BL* | 166 | *IWB2239~IWB6699* | 2.32 | 3.7 | -2.101 |
|  | ZZ2015 | *QaGR.cas-3BL.1* | 122 | *IWB60906~IWB32722* | 2.03 | 6.6 | -2.772 |
|  | BJ2016 | *QaGR.cas-3AS.1* | 35 | *IWB7136~IWA8100* | 2.95 | 4.5 | -1.636 |
|  |  | *QaGR.cas-3DL* | 91 | *IWB52937~IWB17930* | 3.71 | 5.7 | -1.388 |
|  |  | *QaGR.cas-4AL.2* | 79 | *IWB830~IWB1522* | 3.77 | 8.1 | -1.788 |
|  |  | *QaGR.cas-4AL.3* | 139 | *IWB21713~IWB28717* | 3.35 | 5.3 | -1.343 |
|  | Average | *QaGR.cas-2DS.1* | 28 | *IWB41295~IWB60488* | 3.07 | 6.7 | -0.926 |
|  |  | *QaGR.cas-3BL.1* | 118 | *IWB7963~IWB12064* | 2.16 | 3.4 | -0.658 |
|  |  |  |  |  |  | (Contined) | |
| **Trait** | **Environment** | **QTL** | **Position** | **Marker interval** | **LOD** | **PVE** | **Add** |
|  |  | *QaGR.cas-3DL* | 87 | *IWB52937~IWB17930* | 2.88 | 5.6 | -0.835 |
|  |  | *QaGR.cas-6BL.2* | 159 | *IWA4869~IWB28256* | 4.31 | 7.2 | 1.009 |
| Z | ZK2014 | *QaZ.cas-2DS.2* | 48 | *IWB21991~IWB75065* | 2.08 | 3.7 | -0.017 |
|  |  | *QaZ.cas-5AS.2* | 54 | *IWB8074~IWB7316* | 3.12 | 6.3 | 0.023 |
|  |  | *QaZ.cas-7AL.2* | 243 | *IWA4993~IWB12039* | 2.09 | 3.8 | -0.018 |
|  | ZZ2014 | *QaZ.cas-3AS.3* | 109 | *IWB9676~IWB58700* | 2.05 | 3.8 | 0.014 |
|  |  | *QaZ.cas-3BS* | 10 | *IWB11728~IWA5347* | 2.38 | 5.1 | -0.017 |
|  |  | *QaZ.cas-4AS* | 30 | *IWB70645~IWA5858* | 3.32 | 11.5 | -0.025 |
|  |  | *QaZ.cas-5BL.2* | 146 | *IWB71849~IWB73643* | 2.00 | 4.3 | -0.015 |
|  |  | *QaZ.cas-6BL.2* | 185 | *IWB57192~IWB44671* | 2.47 | 5.2 | 0.017 |
|  | ZZ2015 | *QaZ.cas-1BL* | 76 | *IWB22510~IWB27264* | 2.11 | 3.9 | 0.016 |
|  |  | *QaZ.cas-1DS* | 59 | *IWB59650~IWB10694* | 2.13 | 3.5 | -0.015 |
|  |  | *QaZ.cas-2DS.2* | 46 | *IWB21991~IWB75065* | 3.30 | 6.6 | -0.020 |
|  |  | *QaZ.cas-3BL.2* | 167 | *IWB53203~IWB1543* | 3.22 | 6.4 | 0.020 |
|  | BJ2016 | *QaZ.cas-3DL* | 91 | *IWB52937~IWB17930* | 3.57 | 6.2 | -0.030 |
|  |  | *QaZ.cas-4AS* | 43 | *IWB3572~IWB11606* | 2.09 | 6.4 | -0.030 |
|  |  | *QaZ.cas-4AL.2* | 71 | *IWB52955~IWB830* | 2.64 | 4.6 | -0.025 |
|  |  | *QaZ.cas-4AL.3* | 139 | *IWB21713~IWB28717* | 2.06 | 3.5 | -0.022 |
|  | Average | *QaZ.cas-1AL.2* | 108 | *IWB10475~IWB12600* | 3.80 | 5.8 | 0.012 |
|  |  | *QaZ.cas-3AS.2* | 84 | *IWB63999~IWA3939* | 4.39 | 6.9 | -0.013 |
|  |  | *QaZ.cas-4AS* | 49 | *IWA1137~IWB2177* | 4.20 | 10.3 | -0.016 |
|  |  | *QaZ.cas-5AL* | 181 | *IWA3704~IWB48788* | 3.64 | 5.5 | 0.012 |
| FCGR | ZK2014 | *QaFCGR.cas-1AL.1* | 49 | *IWB36443~IWB55507* | 2.04 | 3.4 | 1.160 |
|  |  | *QaFCGR.cas-2DS.2* | 51 | *IWB12962~IWB11197* | 2.33 | 5.8 | -1.511 |
|  |  | *QaFCGR.cas-3AS.3* | 112 | *IWA7022~IWA5151* | 4.03 | 6.6 | 1.615 |
|  |  | *QaFCGR.cas-3DL* | 56 | *IWA5030~IWB34976* | 2.31 | 3.6 | -1.198 |
|  |  | *QaFCGR.cas-6BL.2* | 159 | *IWA4869~IWB28256* | 3.27 | 5.3 | 1.551 |
|  | ZZ2014 | *QaFCGR.cas-2DS.2* | 50 | *IWB12962~IWB11197* | 2.48 | 4.0 | -1.207 |
|  |  | *QaFCGR.cas-4AL.1* | 60 | *IWB27577~IWA4079* | 3.38 | 6.0 | -1.469 |
|  | ZZ2015 | *QaFCGR.cas-2DS.2* | 39 | *IWB42663~IWB21362* | 2.58 | 7.9 | -1.545 |
|  |  | *QaFCGR.cas-3BL.1* | 122 | *IWB60906~IWB32722* | 2.32 | 7.0 | -1.461 |
|  |  | *QaFCGR.cas-5DS* | 20 | *IWB60953~IWB44516* | 2.02 | 3.6 | -1.042 |
|  |  | *QaFCGR.cas-6BS* | 79 | *IWB45294~IWB9751* | 2.51 | 4.7 | 1.197 |
|  | BJ2016 | *QaFCGR.cas-3AS.1* | 35 | *IWB7136~IWA8100* | 2.69 | 4.1 | -1.852 |
|  |  | *QaFCGR.cas-3DL* | 91 | *IWB52937~IWB17930* | 4.96 | 7.5 | -1.901 |
|  |  | *QaFCGR.cas-4AL.2* | 79 | *IWB830~IWB1522* | 3.43 | 7.2 | -2.004 |
|  |  | *QaFCGR.cas-4AL.3* | 139 | *IWB21713~IWB28717* | 4.71 | 7.4 | -1.879 |
|  |  | *QaFCGR.cas-5AS.2* | 55 | *IWB8074~IWB7316* | 2.18 | 4.2 | -1.414 |
|  |  | *QaFCGR.cas-7BL* | 166 | *IWB2239~IWB6699* | 2.18 | 3.1 | -1.221 |
|  | Average | *QaFCGR.cas-2DS.2* | 51 | *IWB12962~IWB11197* | 4.99 | 13.6 | -1.401 |
|  |  | *QaFCGR.cas-3BS* | 12 | *IWB11728~IWA5347* | 2.15 | 3.4 | -0.731 |
|  |  | *QaFCGR.cas-3DL* | 82 | *IWB52937~IWB17930* | 3.74 | 8.3 | -1.099 |
|  |  | *QaFCGR.cas-4B* | 112 | *IWB72706~IWB36208* | 2.46 | 3.8 | -0.768 |
|  |  | *QaFCGR.cas-6BL.2* | 159 | *IWA4869~IWB28256* | 3.91 | 6.4 | 1.029 |

LOD, logarithm of odds score; PVE, percentage of phenotypic variance explained by the QTL; ADD, additive effect of resistance allele; L, long chromosome arms; S, short chromosome arms; ZK, Zhoukou; ZZ, Zhengzhou; BJ, Beijing; MGT, mean germination time; MGR, mean germination rate; GI, weighted germination index; GR, germination ratio; Z, the synchrony index; FCGR, first count germination ratio.
