# Supplementary material for: Genome-Wide Linkage Mapping Reveals QTLs for Seed Vigor-Related Traits Under Artificial Aging in Common Wheat (Triticum aestivum)
Source: Front Plant Sci. 2018 Jul 27;9:1101. doi: 10.3389/fpls.2018.01101 (PMC6073742; doi:10.3389/fpls.2018.01101)
Supplement: Supplementary file 2 [file Table_2.docx]

**Table S2 | Stable QTLs and candidate genes identified in different environments for seed vigor-related traits under artificial aging treatment in RILs**

| **QTL** | **Position**  **(cM)** | **Candidate gene annotation** |
| --- | --- | --- |
| *QaMGT.cas-2DS.2* | 46~51 | Starch synthase 3 |
| *QaMGT.cas-4AS* | 28~44 | Stem rust resistance protein Rpg1, NBS-LRR resistance-like protein |
| *QaMGR.cas-2DS.2* | 47~51 | Starch synthase 3 |
| *QaMGR.cas-4AS* | 28~33 | Stem rust resistance protein Rpg1 |
| *QaGI.cas-3DL* | 57~91 | Dolichyl-diphosphooligosaccharide-protein glycosyltransferase，glutaminyl-peptide cyclotransferase-like, wheat alpha-Amy2/53 gene |
| *QaGR.cas-3DL* | 87~91 | Glutaminyl-peptide cyclotransferase-like, wheat alpha-Amy2/53 gene |
| *QaGR.cas-6BL.2* | 159 | *Aegilops tauschii* protein Kinesin light chain-related 2-like |
| *QaZ.cas-4AS* | 30~49 | Stem rust resistance protein Rpg1, NBS-LRR resistance-like protein |
| *QaFCGR.cas-2DS.2* | 50~51 | Starch synthase 3 |
| *QaFCGR.cas-3DL* | 56~91 | Dolichyl-diphosphooligosaccharide-protein glycosyltransferase，glutaminyl-peptide cyclotransferase-like, wheat alpha-Amy2/53 gene |
