# Supplementary material for: Genome-Wide Linkage Mapping Reveals QTLs for Seed Vigor-Related Traits Under Artificial Aging in Common Wheat (Triticum aestivum)
Source: Front Plant Sci. 2018 Jul 27;9:1101. doi: 10.3389/fpls.2018.01101 (PMC6073742; doi:10.3389/fpls.2018.01101)
Supplement: Supplementary file 3 [file Image_1.PDF]

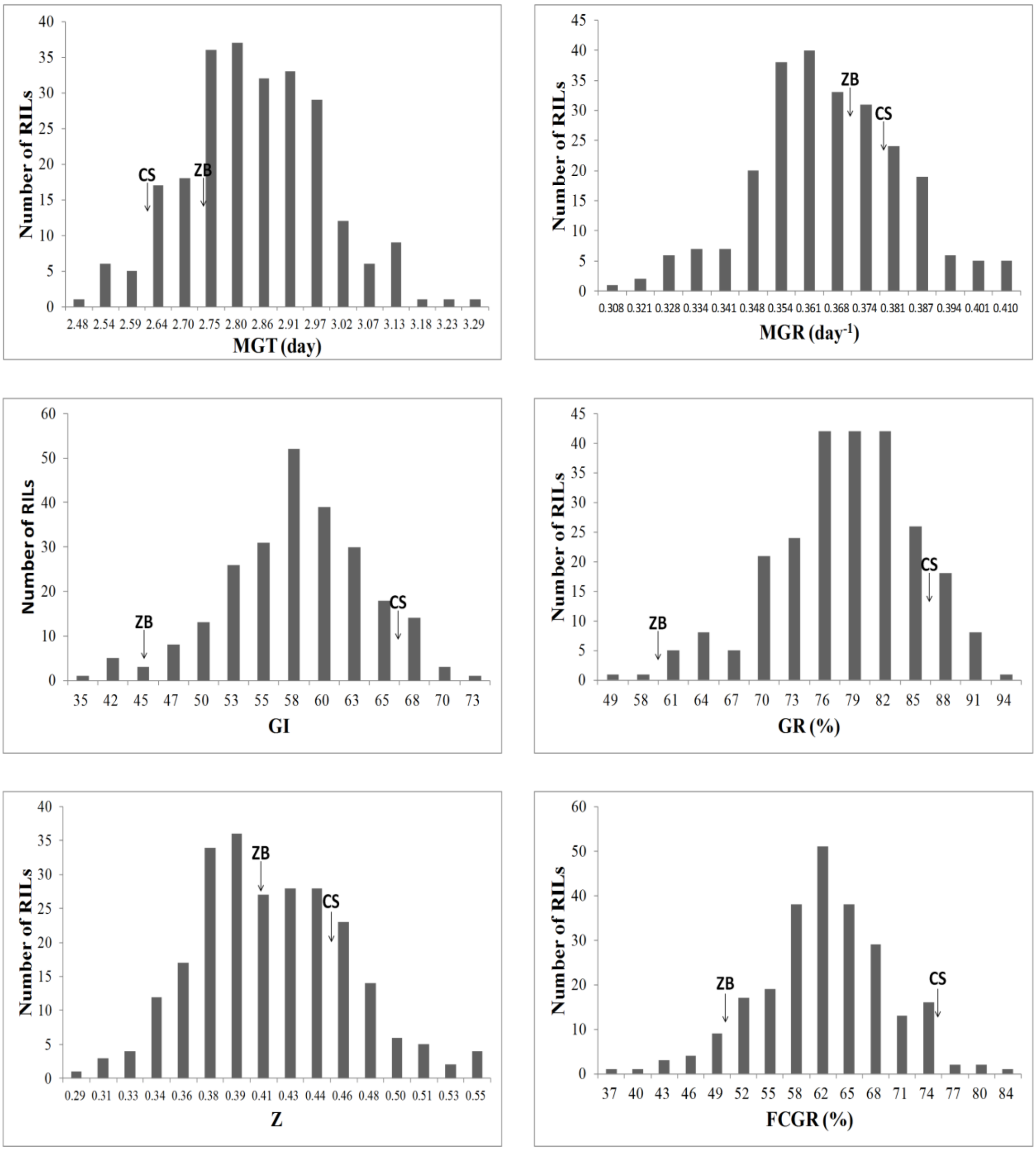

Figure S1 | Frequency distributions of MGT, MGR, GI, GR, Z, and FCGR in the germination tests of CDT.

Arrows represent the parental line. CS, the paternal cultivar Chinese Spring; ZB, the maternal cultivar Zhou 8425B; MGT, mean germination time; MGR, mean germination rate; GI, weighted germination index; GR, germination ratio; Z, the synchrony index; FCGR, first count germination ratio.
